# Health-Related Quality of Life in Burn Injury Victims Attending a Specialized Institution

**DOI:** 10.7759/cureus.106895

**Published:** 2026-04-12

**Authors:** Tania Akter, Sifat Sharmin, Fahmida F Shama, Rezwana P Nisa, Mimuna A Mishu, Fahmida Khanam

**Affiliations:** 1 Emergency Department: Operating Theater, Dhaka Medical College Hospital, Dhaka, BGD; 2 Public Health and Hospital Administration, First Step Development and Research Center, Dhaka, BGD; 3 Preventive and Social Medicine, The National Institute of Preventive and Social Medicine, Dhaka, BGD; 4 Medicine, Mahidol-Oxford Tropical Medicine Research Unit (MORU), Bangkok, THA; 5 Public Health, International Centre for Diarrhoeal Disease Research, Bangladesh (ICDDR, B), Dhaka, BGD; 6 Virology, National Institute of Preventive Medicine (NIPSOM), Dhaka, BGD

**Keywords:** bangladesh, burn injury, burn-specific health scale–brief, health-related quality of life, rehabilitation

## Abstract

Introduction

Burn injuries cause long-term physical, psychological, and social consequences that substantially affect health-related quality of life (HRQOL), particularly in LMICs. The evidence from Bangladesh remains limited. This study aimed to evaluate HRQOL among adult burn injury victims attending a specialized institute and to examine sociodemographic and clinical factors associated with HRQOL.

Methods

This cross-sectional study was conducted between July and December 2023 at the Sheikh Hasina National Institute of Burn and Plastic Surgery, Dhaka. A simple random sampling technique using a daily lottery was employed to recruit 356 eligible inpatients and outpatients aged 18 years or older who were at least 4 weeks post-injury and who fulfilled the predefined inclusion and exclusion criteria. HRQOL was measured via the Bangla-translated Burn Specific Health Scale-Brief (BSHS-B). Sociodemographic and burn-related variables were obtained through face‒to-face interviews. The data were analyzed with SPSS version 23 (IBM Corp., Armonk, NY, US) via descriptive statistics and Mann-Whitney and Kruskal-Wallis tests.

Results

The mean total HRQOL score was 98.96 ± 19.20. Lower scores were observed for work-related functioning, simple abilities, hand function, and heat sensitivity, whereas interpersonal relationships demonstrated comparatively higher scores. HRQOL was significantly associated with age, marital status, and place of residence. Greater total body surface area burned, burn-related complications, wearing synthetic or woollen clothing, multiple surgical procedures, longer hospital stays, and outdoor burn incidents were also linked to lower HRQOL scores. No significant associations were found with sex, monthly family income, or cause of burn.

Conclusions

Burn injury survivors experience substantial impairment across several HRQOL domains, particularly those related to occupational and functional capacity. Strengthening long-term rehabilitation, psychosocial support, and decentralized burn-care services is essential to improve postburn outcomes in resource-limited settings.

## Introduction

Burn injuries represent a significant global public health concern, causing profound morbidity and mortality, particularly in low- and middle-income countries (LMICs) [1,2]. The World Health Organization estimates that burn injuries account for approximately 180,000 deaths annually, with a disproportionately high incidence in LMICs [2,3]. In addition to immediate mortality, burn trauma leads to permanent physical disfigurement, disability, and psychological distress, significantly impacting survivors' long-term health-related quality of life (HRQoL) [4-6].

The impact of burn injuries extends beyond the initial trauma, creating a dynamic and evolving process that affects every aspect of a patient's life [4]. However, despite advancements in acute burn care, a critical gap persists in the understanding and management of long-term sequelae, particularly concerning HRQoL [4,7].

HRQoL is a multidimensional concept encompassing physical, psychological, social, and functional well-being, and its assessment is crucial for evaluating the true burden of disease and the effectiveness of therapeutic interventions [4,5]. For burn survivors, factors such as persistent pain, anxiety, depression, social appearance anxiety, and body perception issues are commonly reported, profoundly diminishing their HRQoL [6,7]. Studies have highlighted the prevalence of psychosocial compromise in survivors of extensive burns, emphasizing the need to understand injury and demographic-related factors that predispose individuals to such outcomes [7]. The challenges faced by burn patients in LMICs are often exacerbated by economic hardships, vocational limitations, and social exclusion, leading to increased stigma and discrimination within healthcare settings and society [8]. This context further compounds mental health concerns among survivors [8].

In Bangladesh, burn injuries are prevalent, and a detailed analysis of their epidemiological characteristics and factors influencing severity is crucial [9,10]. The South Asian region, including Bangladesh, contributes approximately 59% of the global mortality due to burns, yet comprehensive literature on outcomes from these LMICs is scarce [11]. Efforts to improve burn injury data collection and the quality of databanks, such as the South Asian Burn Registry (SABR) project, are essential for meaningful epidemiological studies and targeted interventions [11,12]. The lack of updated and valid data also extends to injury-related disabilities, which remain a neglected public health agenda in Bangladesh [13].

Given the substantial physical, psychological, and socioeconomic consequences of burn injuries, especially in a resource-limited setting, such as Bangladesh, it is imperative to assess the HRQoL of burn injury victims comprehensively. While general HRQoL studies have been conducted in Bangladesh that focus on conditions such as hypertension and chronic obstructive pulmonary disease [14,15], among specific populations such as *madrasa* students [16], dedicated research specifically addressing burn injury survivors is urgently needed. Research indicates that exercise programs can improve the quality of life of burn patients [17], and family-based education and follow-up programs aim to enhance psychosocial outcomes for patients and their families [18]. However, the experiences of burn therapists themselves highlight the high levels of anxiety and stress due to patient exposure to traumatic events, which impacts their own professional quality of life [19]. Therefore, the primary objective of this study was to assess HRQOL among adult burn injury victims attending a specialized institute in Dhaka, Bangladesh. The secondary objective was to identify sociodemographic factors, including age, sex, marital status, and residence, and clinical factors, including total body surface area burned, complications, number of surgeries, length of hospital stay, type of clothing, and place of burn, associated with HRQOL.

## Materials and methods

Study setting and study population

This cross-sectional study was conducted between July 1 and December 31, 2023, among burn victims attending the Sheikh Hasina National Institute of Burn and Plastic Surgery, Dhaka. Participants were selected using simple random sampling through a daily lottery method. During the data collection period, eligible patients from both inpatient wards and the outpatient department were screened daily according to predefined inclusion and exclusion criteria. A daily sampling frame was prepared by listing all eligible patients from hospital admission registers and outpatient attendance records. Each eligible patient was assigned a serial number, and simple random selection was performed using a lottery method in which numbered slips were mixed thoroughly and randomly drawn to recruit participants for that day. Each draw was conducted by a trained data collector under supervision to ensure randomness and transparency, and no replacement was allowed once a participant was selected for that day. Patients already enrolled in the study were excluded from subsequent daily listings to prevent duplication. Individuals aged 18 years or older with a burn injury duration of at least four weeks were eligible for inclusion. Patients with preexisting psychiatric illness and those who were severely ill or admitted to the intensive care unit or high-dependency unit were excluded from the study. As the study was conducted in a single specialized center, the possibility of selection bias cannot be excluded.

The sample size was calculated via the single-population mean formula:

\[ n = \left(\frac{Z_{\alpha/2} \times \sigma}{d}\right)^2 \]

where n denotes the required sample size, Z is 1.96 for a 95% confidence level, σ is the assumed standard deviation of the HRQOL score (25) derived from a previous study [20], and d represents the margin of error. A relative precision of 5% was applied based on the expected mean HRQOL score of 140 [20]; therefore, \[ d = 0.05 \times 140 = 7 \]

Accordingly, the minimum required sample size was calculated as:

\[ n = \left(\frac{1.96 \times 25}{7}\right)^2 = 49 \]

After allowing for a 10% anticipated nonresponse rate, the final estimated sample size was 55. Although the minimum calculated sample size was 55, a total of 356 participants were included in the study to increase the statistical precision and representativeness.

Data collection tools and procedures

Data were collected using a structured Burn-Specific Health Scale-Brief (BSHS-B) questionnaire, a disease-specific instrument designed to assess HRQOL among burn patients. The scale comprises 9 domains with a total of 40 items, including affect, heat sensitivity, hand function, treatment regimen, work, sexuality, interpersonal relationships, simple abilities, and body image [21,22]. Each item was scored on a five-point Likert scale (0-4), where 0 indicated extreme difficulty, and 4 indicated no difficulty, with higher scores reflecting better quality of life.

The questionnaire was translated into Bangla following a standardized forward-backward translation process. Initially, the original English version was independently translated into Bangla by two bilingual experts. A reconciled Bangla version was then back-translated into English by another independent translator who was blinded to the original questionnaire. Discrepancies between the original and back-translated versions were reviewed and resolved by a panel of experts to ensure conceptual and semantic equivalence. A pretest was conducted among 5% of the study population to assess the clarity, comprehensibility, and cultural appropriateness of the translated instrument. Participants included in the pretest were excluded from the final analysis.

Data were collected through face-to-face interviews conducted by the researcher, during which the questionnaire was explained to each participant to ensure proper understanding. Domain scores were calculated as summed scores, and the results were presented as mean and standard deviation. A higher overall score indicated better HRQOL [21,22].

The internal consistency reliability of the BSHS-B was assessed using Cronbach’s alpha, which demonstrated excellent reliability (α = 0.93). The total body surface area (TBSA) affected by burns was determined using the Lund and Browder chart [23]. Information regarding the place of burn was obtained through participant interviews and review of medical records. The place of burn was categorized into a domestic setting and an outdoor setting. The domestic setting referred to burn injuries occurring within household premises, such as the kitchen, bedroom, living area, or courtyard, whereas the outdoor setting referred to injuries occurring outside the household, including the workplace, road, industrial area, or other external environments. The BSHS-B is a widely used instrument and was utilized in this study for academic and non-commercial purposes without modification to its original structure.

Data analysis

Data were checked for completeness prior to analysis. Cases with incomplete or missing responses were excluded from the respective analyses, and no imputation was performed. The data were analyzed using SPSS version 23 (IBM Corp., Armonk, NY, US). Descriptive statistics were computed, and the results are presented as frequencies, percentages, means, and standard deviations through tables and graphs. The BSHS-B score was calculated and summarized as the minimum, maximum, mean, and standard deviation for each domain. The normality of the data was assessed using the Shapiro-Wilk test. As the HRQOL scores were not normally distributed, non-parametric tests were applied for group comparisons. Sociodemographic and burn-specific variables were analyzed against the mean total score and domain scores using the Mann-Whitney U test and the Kruskal-Wallis test, as appropriate. A p-value of less than 0.05 was considered statistically significant.

Ethical considerations

Ethical approval was obtained from the Institutional Review Board of the National Institute of Preventive and Social Medicine (NIPSOM), Dhaka, Bangladesh (Approval no: NIPSOM/IRB/2023/06). Informed consent was also obtained from the participants before data collection.

## Results

Sociodemographic characteristics

The mean age of the participants was 32.49 ± 11.23 years (range: 18-65 years). The majority of the respondents were aged 18-32 years (206; 57.9%), 227 (63.8%) were male, and 277 (77.8%) were married. Among the participants, 201 (56.5%) resided in rural areas. In terms of education, 125 (35.1%) had completed secondary education, whereas 39 (11%) had attained graduate-level or higher education. The monthly family income ranged from 6,000 to 18,000 BDT for 222 (62.4%) respondents. Detailed socio-demographic characteristics are presented in Table 1.

**Table 1:** Socio-demographic characteristics of burn injury victims (n=356) BDT: Bangladeshi taka

Variable	Category	Frequency	Percentage (%)
Age (years)	Mean ± SD	32.49 ± 11.23	
	18–32	206	57.9
	33–65	150	42.1
Gender	Male	227	63.8
	Female	129	36.2
Marital status	Married	277	77.8
	Unmarried	79	22.2
Place of residence	Urban	155	43.5
	Rural	201	56.5
Level of education	Illiterate	38	10.7
	Primary	102	28.7
	Secondary	125	35.1
	Higher secondary	52	14.6
	Graduate or above	39	11.0
Monthly family income (BDT)	6,000–18,000	222	62.4
	19,000–50,000	134	37.6

Clinical characteristics

Regarding clinical attributes of the burn injury victims, 182 (51.1%) participants did not receive first aid after the burn incident, while 174 (48.9%) received some form of first aid. Cotton clothing was worn by 186 (52.2%) participants at the time of injury. Flame burns were the most common cause, affecting 148 (41.6%) participants, followed by electric burns in 134 (37.6%), scald burns in 67 (18.8%), and chemical burns in 7 (2.0%). Most participants, 315 (88.5%), had burns involving 10-25% TBSA. Burn-related complications occurred in 215 (60.4%) cases. Most patients underwent one or two surgeries, and 162 (45.5%) had a hospital stay of 1-2 months (Table 2).

**Table 2:** Clinical attributes of burn injury victims (n=356) TBSA: Total body surface area

Variables	Category	Frequency	Percentage (%)
Taking first aid	Yes	174	48.9
	No	182	51.1
Types of clothing	Cotton	186	52.2
	Synthetic	162	45.5
	Woolen	8	2.2
Causes of burn	Flame burn	148	41.6
	Electric burn	134	37.6
	Chemical burn	7	2.0
	Scald burn	67	18.8
Percentage of TBSA	10%-25%	315	88.5
	26-40%	41	11.5
Complications of burn	Yes	215	60.4
	No	141	39.6
Number of surgeries faced	1 time	95	26.7
	2 times	159	44.7
	> 3 times	38	10.7
	No surgery	64	18.0
Length of stay in the hospital	<1 month	101	28.4
	1-2 months	162	45.5
	2-4months	83	23.3
	>4 months	10	2.8

HRQOL

The mean summed domain scores indicated that participants scored lowest in the work and economic impact domain (4.7 ± 2.5) and highest in the interpersonal relationship domain (14.7 ± 2.9) (Table 3).

**Table 3:** Distribution of respondents by mean HRQOL score (n=356) HRQOL: Health-related quality of life

Burn Specific Health Scale-Brief (BSHS-B) domains	Mean (SD) of summed domain scores
1. Simple abilities and mobility (3 items)	5.4 (3.4)
2. Hand function (5 items)	9.7 (5.4)
3. Heat sensitivity (5 items)	8.0 (4.4)
4. Treatment regimen (5 items)	16.8 (2.8)
5. Affect (7 items)	19.7 (5.1)
6. Body image (4 items)	12.03 (4.2)
7. Interpersonal relationship (4 items)	14.7 (2.9)
8. Work and economic impact (4 items)	4.7 (2.5)
9. Sexuality (3 items)	10.8 (2.3)
Overall HRQOL score	98.96 ± 19.20

Associations between HRQOL and socio-demographic/clinical variables

Significant associations between BSHS-B scores and several sociodemographic and clinical variables were observed. Younger participants aged 18-32 years demonstrated lower BSHS-B scores compared with older individuals, with a small effect size (r = 0.14, p = 0.007). Marital status (r = 0.13, p = 0.015) and place of residence (r = 0.14, p = 0.010) also showed significant associations, indicating small effects. Among clinical variables, percentage of TBSA burned (r = 0.18, p = 0.001), place of burn (r = 0.22, p < 0.001), and presence of complications (r = 0.24, p < 0.001) demonstrated small to moderate effect sizes. Types of clothing (η² = 0.011, p = 0.032), number of surgeries (η² = 0.025, p = 0.009), and length of hospital stay (η² = 0.005, p = 0.038) showed small but significant associations with BSHS-B scores. No significant association was observed with gender, family monthly income, or causes of burn (Table 4).

**Table 4:** Associations between BSHS-B scores and sociodemographic and clinical characteristics BDT: Bangladeshi taka; BSHS-B: Burn-Specific Health Scale-Brief

Variable	Category	n	Mean rank	Test statistic	Effect size	P-value
Age group	18–32 years	206	161.34	U = 12087.0, Z = -2.680	r = 0.14	0.007
	33–65 years	150	190.56			
Gender	Male	227	167.14	U = 12352.5, Z = -1.582	r = 0.09	0.114
	Female	129	184.88			
Place of residence	Urban	155	189.35	U = 12323.0, Z = 2.579	r = 0.14	0.010
	Rural	201	161.37			
Marital status	Married	277	181.13	U = 9382.5, Z = 2.431	r = 0.13	0.015
	Unmarried	79	151.12			
Family monthly income (BDT)	6,000–18,000	222	172.34	U = 4547.5, Z = -0.687	r = 0.04	0.492
	19,000–50,000	134	185.27			
Causes of burn	Flame burn	148	172.14	H = 7.398, df = 3	η² = 0.013	0.060
	Electrical burn injury	134	160.55			
	Chemical burn	7	208.64			
	Scald burn	67	199.65			
Percentage of TBSA	10%–25%	315	180.03	U = 3983.0, Z = 3.406	r = 0.18	0.001
	26%–40%	41	122.13			
Place of burn	Domestic setting	195	193.69	U = 11057.0, Z = 4.096	r = 0.22	<0.001
	Outdoor	161	149.48			
Types of clothing	Cotton	186	184.39	H = 5.906, df = 2	η² = 0.011	0.032
	Synthetic	162	163.50			
	Woolen	8	117.71			
Complications	Yes	215	153.51	U = 10248.5, Z = -4.538	r = 0.24	<0.001
	No	141	203.27			
Number of surgeries	1 time	95	181.58	H = 11.688, df = 3	η² = 0.025	0.009
	2 times	159	162.35			
	>3 times	38	146.09			
	None	64	205.35			
Length of hospital stay	<1 month	101	185.79	H = 4.669, df = 3	η² = 0.005	0.038
	1–2 months	162	161.17			
	2–4 months	83	182.75			
	>4 months	10	162.50			

## Discussion

This study examined HRQOL among adult burn injury victims attending a specialized institute in Dhaka. Overall, survivors showed marked HRQOL impairment, with the greatest deficits in work/economic impact and functional domains, while interpersonal relationships were comparatively preserved. However, poorer HRQOL clustered among younger, unmarried, rural patients, and those with greater burn severity and complications.

This study revealed associations between sociodemographic variables and HRQoL, which aligns with patterns reported in the international literature, particularly from LMICs. Younger individuals (18-32 years) reported significantly lower HRQoL than older adults did, a finding that may reflect greater vocational and social disruption during critical developmental stages, as well as prolonged exposure to stigma and limitations [8]. Unmarried respondents also experienced poorer HRQoL, possibly indicating a lack of social support systems that married individuals might benefit from, which is a crucial factor in postburn psychological adjustment [18]. The disparity between rural and urban residents, with rural inhabitants exhibiting lower HRQoL, can be attributed to several factors, including limited access to specialized burn care, rehabilitation services, and socioeconomic opportunities in rural areas, mirroring challenges identified in rural northern Bangladesh, where access to care is restricted [24,25].

Clinical factors were also robust predictors of HRQoL. A greater TBSA burned, and the presence of burn-related complications, was significantly linked to a lower HRQoL. This finding is consistent with extensive literature demonstrating that burn severity directly correlates with long-term physical limitations, pain, and psychological distress [4,5]. The number of surgeries and length of hospital stay also showed significant relationships with domain-specific HRQoL scores, reflecting the prolonged recovery period and potential for complications associated with severe burns, which profoundly impact daily functioning and mental well-being [4]. Interestingly, participants who wore synthetic or woollen clothing at the time of injury demonstrated poorer HRQoL. This finding may be related to the increased severity and depth of burns associated with such materials, although this study did not directly assess burn mechanisms [4]. The finding that burns occurring in domestic settings were associated with a greater quality of life than those occurring in outdoor incidents suggests potential differences in injury mechanisms, immediate care received, or perhaps the social support structures available within the domestic environment, although further research is needed to fully elucidate this relationship. This difference may reflect variations in injury context or access to immediate care; however, this was not directly evaluated in the present study [24].

The HRQoL domain patterns, particularly the lowest scores for work and economic impact and the highest scores for interpersonal relationships, offer important insights. The severe impact on work and economic aspects is a pervasive issue for burn survivors globally, stemming from physical disabilities, disfigurement, and societal stigma that hinder employment opportunities [8]. This is especially pertinent in LMICs, where social safety nets are often inadequate. Conversely, higher scores in interpersonal relationships may suggest the resilience of family and community bonds in Bangladesh, providing a critical buffer against the broader negative impacts of burn injuries.

Notably, sex and the cause of burn (e.g., flame, electric) did not significantly influence HRQoL in this study. While some studies in other contexts have reported gender differences in HRQoL following burns, often related to societal roles or access to care, this finding suggests that in this specific Bangladeshi context, the burden of burn injury might be more universally felt across genders or that other factors exert a stronger influence on HRQoL [24]. Similarly, the lack of a significant association with the specific cause of burn, despite the prevalence of flame and electric burns [12], might indicate that the overall severity and consequences of the burn, rather than its etiology, are the primary drivers of long-term HRQoL outcomes.

This study benefits from a relatively large sample size (n=356) from a specialized institute in Dhaka and the use of a validated, disease-specific HRQoL assessment tool (BSHS-B) with excellent internal consistency (Cronbach’s α = 0.93), which enhances the reliability of the findings. The inclusion of both inpatient and outpatient participants also improves the comprehensiveness of the study population. However, several limitations must be acknowledged. The cross-sectional design precludes the establishment of causal relationships between identified factors and HRQoL outcomes. As a single-center study, the findings may not be fully representative of the entire burn survivor population in Bangladesh, particularly those in remote or resource-limited settings. Furthermore, reliance on self-reported data introduces the possibility of recall and reporting bias. There is also a potential for selection bias, as the study population was facility-based.

## Conclusions

The present study demonstrated that burn injuries are associated with impaired HRQOL, with the greatest deficits in occupational, functional, and heat-sensitivity domains. Poorer HRQOL was associated with younger age, unmarried status, rural residence, higher TBSA involvement, complications, prolonged hospitalization, multiple surgeries, and synthetic clothing exposure. Long-term multidisciplinary rehabilitation and decentralized burn-care services are recommended, and future longitudinal studies are warranted to clarify causal pathways and recovery trajectories.
